# Supplementary material for: Nanoarchitectonics of Three-Dimensional Carbon Nanofiber-Supported Hollow Copper Sulfide Spheres for Asymmetric Supercapacitor Applications
Source: Int J Mol Sci. 2023 Jun 2;24(11):9685. doi: 10.3390/ijms24119685 (PMC10253980; doi:10.3390/ijms24119685)
Supplement: Supplementary file 1 [file ijms-24-09685-s001.zip › ijms-2415636-supplementary.pdf]

Nanoarchitectonics of Three-Dimensional Carbon Nanofibers Supported Hollow Copper Sulfide Spheres for Asymmetric Supercapacitor Applications

Miyeon Shin[a#], Ganesh Prasad Awasthi [b#], Krishna Prasad Sharma [b]-Puran Pandey [c], Mira Park[d], Gunendra Prasad Ojha,[d]*, Changho Yu [a, b]*

[a]*Department of Energy Storage/Conversion Engineering of Graduate School, Jeonbuk National University, Jeonju, Jeollabuk-do, 54896, Republic of Korea*

[b]*Division of Convergence Technology Engineering, Jeonbuk National University, Jeonju, Jeollabuk-do, 54896, Republic of Korea*

[c]*Division of Physics and Semiconductor Science, Dongguk University-Sepul, Seoul 04620, Republic of Korea*

[d]*Carbon Composite Energy Nanomaterials Research Center, Woosuk University, Republic of Korea*

# *These authors contributed equally to this work.*

[*]Corresponding authors

Changho Yu, Ph.D.

E-mail: goody0418@jbnu.ac.kr

Gunendra Prasad Ojha, Ph.D.

E-mail: gpojha10@gmail.com

E-mail: gpojha10@gmail.com

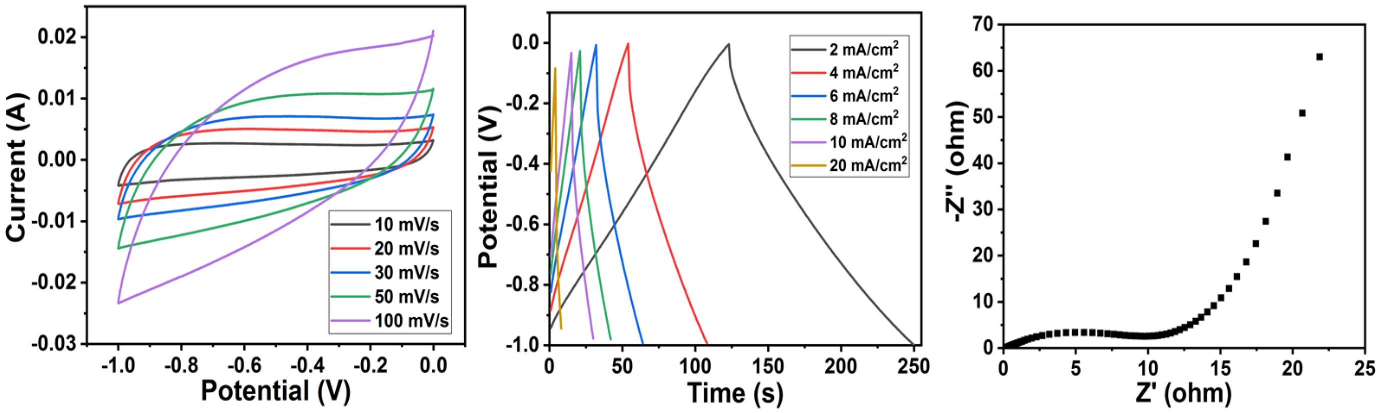

(a) (b) (c)

Figure S1: Electrochemical performance of (BAC); The cyclic voltammetry curves of different scan rates (10 – 100 mV/s) (a), gravimetric charge discharge curves of different current densities (2-10 mA/cm^2) (b) and Nyquist plot of EIS (c) at 2M KOH electrolyte solution, respectively.
